# Adult patients’ experiences of patient-professional communication in patient portals: a qualitative systematic review protocol

**DOI:** 10.11124/JBIES-21-00091

**Published:** 2021-11-24

**Authors:** Moona Huhtakangas, Anna-Maria Tuomikoski, Elina Laukka, Maria Kääriäinen, Outi Kanste

**Affiliations:** 1 Department of Nursing Science and Health Management, University of Oulu, Oulu, Finland; 2 The Finnish Centre for Evidence-Based Health Care: A JBI Centre of Excellence, Helsinki, Finland; 3 Oulu University of Applied Sciences, University of Oulu, Oulu, Finland; 4 Finnish Institute for Health and Welfare, Health and Social Service System Research Team, Helsinki, Finland

**Keywords:** communication, digital health care, experiences, patient portals, systematic review

## Abstract

**Objective:**

The objective of the review is to identify, critically appraise, and synthesize the best available evidence on adult patients’ experiences of patient-professional communication in patient portals.

**Introduction:**

Alongside face-to-face communication, patient portals can improve care quality and patients’ self-management of chronic diseases. It is important to examine how patients experience patient-professional communication in patient portals because this digital environment inherently lacks non-verbal messages, which can lead to misunderstandings.

**Inclusion criteria:**

Qualitative studies that describe patients’ experiences of reciprocal patient-professional communication in patient portals will be included. Patients must be over the age of 18 years and have a need for long-term care delivered by a health care professional (eg, patients with chronic diseases, such as cancer or diabetes). The health care professionals considered for inclusion are the members of the patient's health care team who communicate with the patient using patient portals. A patient portal is defined as a personal health record, which is either an independent webpage or interconnected with an electronic health record.

**Methods:**

The following databases will be searched: MEDLINE (PubMed), CINAHL (EBSCO), ProQuest (Abi/Inform), Scopus, Medic, Google Scholar, Science Direct and Cochrane CENTRAL. Gray literature will be searched in MedNar. Studies published in English, Finnish, or Swedish will be considered, and there is no date limitation. Studies will be screened and critically appraised for methodological quality by two independent researchers. Data will be extracted using a standardized tool from JBI SUMARI. Data synthesis will be conducted according to the meta-aggregation approach. Confidence in the evidence will be assessed using the ConQual approach.

**Systematic review registration number:**

PROSPERO CRD42021286177

## Introduction

Effective communication between patients and health care professionals has been linked to higher patient satisfaction,^1^ and is one of the key characteristics of patient-centered care.^2^ Digitalization in the health care sector has led to a higher incidence of telemedicine, which enables health care providers to provide services from a distance.^3^ Telemedicine interventions include remote consultations between the patient and health care professionals.^4^ Telemedicine services are often provided alongside face-to-face communication. They can be considered complementary to face-to-face health care services because this new form of care can improve patients’ access to care,^5^ patient engagement, and adherence to treatment.^6^

A few examples of telemedicine services are video and phone consultations, emails, text messages, and patient portals. Video consultations have the advantage of including non-verbal messages in the communication process between patients and health care professionals. However, when compared with face-to-face consultations, patients have experienced challenges in asking questions during video consultations, and feel that the professional does not pay as much attention to them. Furthermore, patients have reported difficulties in establishing a relationship with the health care professional.^7^ Unlike video consultations, communication that occurs in patient portals does not include non-verbal messages; thus, even greater emphasis should be given to effective and clear communication in this medium of communication.

A patient portal is defined as a personal health record (owned and administered by a health care organization) that is interconnected with an electronic health record or an independent webpage that is not connected to an electronic health record where patients can access their medical information. Additional features of patient portals include medication information, potential to access laboratory results, and secure messaging with health care providers, which gives patients an additional way to contact the health care team.^8^ Thus, unlike emails and text messages, patient portals are more firmly connected to patients’ coordinated care. While communication in patient portals leaves a trail in patient portals (and can be accessed in the future in relation to other information regarding the patient's care), emails and text messages exchanged between the healthcare professional and the patient are not conveniently accessible. Through patient portals it is also possible to schedule appointments, request medication refills, and update health information before consultations. Patient portals are used globally, mostly in Asia, Europe, the United States, and Australia. For example, in the United Kingdom, United States, and Canada, national digital health programs and strategies have been launched to increase communication between patients and health care professionals through digital health care services, such as patient portals.^9^

There is evidence that patient portals can improve care quality, patients’ self-management of chronic diseases,^10^ and patient-provider communication.^8,10^ A patient's age, education level, health status, and health literacy are some of the previously identified factors associated with a patient's ability and interest in using patient portals. In addition to these patient-specific factors, encouragement from health care professionals and the usability of the portal affect patients’ use of patient portals.^11^ However, in an analysis of the messages exchanged between patients and health care professionals through patient portals, Alpert *et al.*^12^ found that more than half of the replies sent by health care professionals showed a lack of supportive talk and partnership building. Because of the increasing use and adoption of patient portals as part of patient care, it is important to examine these aspects of communication in order to develop patient portals that enable more efficient communication.

Stewart *et al.*^13^ previously presented a conceptual framework for patient-professional communication. This framework takes into account the reciprocal nature of communication between the patient and the health care professional. According to the first component of the framework, communication enables both participants (patient and health care professional) to achieve their goals, which are a concrete expression of their needs; for example, a patient's goal of seeking medical information expresses a desire to gain evidence that can be used to make a decision about treatment. In addition, the framework considers how participants’ emotions, needs, skills, values, and beliefs influence communication. These factors affect how the participants form the messages they convey, and how they understand the messages they receive. The third component describes the communication process (ie, conveying and receiving messages). More specifically, messages may be sent either intentionally or unintentionally, and can be either verbal or non-verbal (eg, tone of voice, body language). It is also important to note that the communication process includes passive messages (eg, silence when a response is expected, lack of discussion). The last component is environment, which includes the physical setting in which the communication takes place. This component is influenced by factors such as time pressure, along with factors that are external to the communication process (eg, new information about the patient's condition).^13^

Using previously published patient-professional communication frameworks developed for face-to-face consultations as a basis, Hong *et al.*^14^ developed a framework for digital tool–enabled patient-provider communication based on digital interventions. The study was conducted using systematic literature reviews, but the framework was developed only for the purposes of a theoretical framework. The presented framework included four components: making shared decisions, enabling self-management, providing emotional or social support, and exchange of information. Although some aspects of face-to-face patient-professional communication can also be identified in digital health care (eg, patient goals), the patient-professional communication framework developed for face-to-face consultations is not always suitable for patient portals (eg, the lack of non-verbal messages in the digital realm). In the framework by Stewart *et al.*^13^ for patient-professional communication, the environment was identified as one of the components to be considered. When care occurs in a digital environment, it is essential to understand which aspects of communication should be considered, given that the basis for interaction differs from traditional face-to-face communication. Research examining which aspects of patient-professional communication are relevant in a digital environment is a prerequisite for the development of robust patient-centered health care services that are also applicable to digital settings.

Previous systematic reviews have examined various aspects of patient portals; for example, their effect on patient engagement^15^; the mediating effect of electronic medical records on communication between professionals, patients, and their families to support engagement in care^16^; and how the use of electronic medical records during face-to-face consultation affects patient-professional communication and relationships.^17^ Moreover, Sakaguchi-Tang *et al.*^18^ examined older adults’ experiences of patient portal use, including the barriers to and benefits of patient portal use. A systematic review^19^ found that, on average, the adoption rate of patient portals by patients was only 52%, indicating a need to understand ways to increase this adoption rate in order to improve care and health through patient portals. In another review, Irizarry *et al.*^11^ examined how patient portals can support patient engagement, while Hong *et al.*^14^ focused on identifying digital interventions that can improve patient-provider communication for cancer patients. Laukka *et al.*^20^ examined patient-professional communication in patient portals from the viewpoint of health care professionals. However, no previous systematic review has covered patients’ experiences of communication with health care professionals using patient portals.

In order to understand the various dimensions of communication between a patient and a health care professional through a patient portal, it is vital to examine patients’ points of view. This understanding gives a basis for defining what aspects are important in patient-professional interaction and patient-centered care when part of that care occurs in a digital environment. Furthermore, the results can be used to develop an instrument measuring patient-professional communication in patient portals. The results can also be used when providing training for health care professionals about effective, patient-centered communication in digital health care. A preliminary search of PROSPERO, MEDLINE, the Cochrane Database of Systematic Reviews and *JBI Evidence Synthesis* was conducted and no current or in-progress systematic reviews on the topic were identified.

The objective of the review is to identify, critically appraise, and synthesize the best available evidence on adult patients’ experiences of patient-professional communication in patient portals.

## Review question

How do adult patients experience patient-professional communication in patient portals?

## Inclusion criteria

### Participants

This review will include patients who recurrently access digital health care services through patient portals. The inclusion criteria for participants are: patients over the age of 18 years who have a need for long-term care (eg, patients with chronic diseases, such as cancer or diabetes). Participants who use patient portal services on a non-recurring basis (ie, they do not need long-term care) will be excluded.

### Phenomena of interest

This review will consider studies that describe patients’ experiences of patient-professional communication in patient portals. Either the patient or the health care professional can contact each other through the patient portal and start the conversation. For the communication to be reciprocal, the patient has to be included in the conversation, thus, studies that only describe one-sided communication will be excluded. Moreover, only patients’ experiences of the studied phenomenon will be included, so studies that have analyzed patient-professional communication based on exchanged messages in patient portals will be excluded. Health care professionals are defined as members of the patient's health care team who communicate with the patient using patient portals.

### Context

The context is patient portals that include reciprocal communication (asynchronous or synchronous) between patients and health care professionals (eg, via secure messaging). Patient portals provided by specialized health care or primary health care services will be included. The following exclusion criteria will be used: free-for-use online platforms where a patient can contact a health care professional (eg, ask-a-doctor platforms) and consultations through video, email, phone, text messages, or apps (eg, WhatsApp). Research will not be excluded based on the country of origin.

### Types of studies

This review will consider studies that focus on qualitative data including, but not limited to, designs such as qualitative descriptive studies, phenomenology, grounded theory, ethnography, action research, and feminist research. The qualitative results of mixed method studies will be considered.

## Methods

The proposed systematic review will be conducted in accordance with JBI methodology for systematic reviews of qualitative evidence.^21^ The review title is registered in PROSPERO (CRD42021286177).

### Search strategy

The search strategy will aim to locate both published and unpublished studies. An initial limited search of PubMed and CINAHL (EBSCO) was undertaken to identify articles on the topic. The text words (ie, free text words) contained in the titles and abstracts of relevant articles identified in the initial search, and the index terms (ie, MeSH terms in MEDLINE [Ovid]) used to describe the articles were used to develop a full search strategy for MEDLINE (Ovid; see Appendix I). The search strategy, including all identified keywords and index terms, will be adapted for each included database and/or information source. The reference list of all included sources of evidence will be screened for additional studies.

Studies published in English, Finnish, or Swedish will be included. Limitations regarding publication date will not be applied.

The databases to be searched include MEDLINE (PubMed), CINAHL (EBSCO), ProQuest (Abi/Inform), Scopus, Medic (a Finnish database), Google Scholar, Science Direct, and Cochrane CENTRAL. MedNar will be searched to identify unpublished studies and gray literature.

### Study selection

Following the search, all identified citations will be collated and uploaded into the Covidence systematic review software package (Veritas Health Innovation, Melbourne, Australia) and duplicates removed. Following a pilot test, titles and abstracts will then be screened by two independent researchers for assessment against the inclusion criteria for the review. Potentially relevant studies will be retrieved in full and their citation details imported into the JBI System for the Unified Management, Assessment and Review of Information (JBI SUMARI; JBI, Adelaide, Australia). The full text of selected citations will be assessed in detail against the inclusion criteria by two or more independent researchers. Reasons for exclusion of papers at full text that do not meet the inclusion criteria will be recorded and reported in the systematic review. Any disagreements that arise between the researchers at each stage of the selection process will be resolved through discussion or with an additional researcher. The results of the search and the study inclusion process will be reported in full in the final systematic review and presented in a Preferred Reporting Items for Systematic Reviews and Meta-Analyses (PRISMA) flow diagram.^22^

### Assessment of methodological quality

Eligible studies will be critically appraised by two independent researchers for methodological quality using the standard JBI critical appraisal checklist for qualitative research.^21^ Authors of papers will be contacted to request missing or additional data for clarification, where required. Any disagreements that arise between the researchers will be resolved through discussion or with a third researcher. The results of critical appraisal will be reported in narrative form and in a table.

Following critical appraisal, studies that do not meet a certain quality threshold will be excluded. This decision will be based on the critical appraisal scores of the studies; more specifically, studies that receive a critical appraisal score of at least 5 out of 10 will be included.

### Data extraction

Data will be extracted from studies included in the review by two independent researchers using the standardized data extraction tool in JBI SUMARI.^21^ The data extracted will include specific details about the populations, context, culture, geographical location, study methods, and the phenomena of interest relevant to the review objective. The findings, and their illustrations, will be extracted and assigned a level of credibility. Any disagreements that arise between the researchers will be resolved through discussion or with a third researcher. Authors of papers will be contacted to request missing or additional data, where required.

### Data synthesis

Qualitative research findings will, where possible, be pooled using JBI SUMARI using the meta-aggregation approach.^23^ This will involve the aggregation or synthesis of findings to generate a set of statements that represent that aggregation, through assembling the findings and categorizing them on the basis of similarity in meaning. These categories will then be synthesized to produce a single comprehensive set of synthesized findings that can be used as a basis for evidence-based practice. Where textual pooling is not possible, the findings will be presented in narrative format. Only unequivocal and credible findings will be included in the synthesis.

### Assessing confidence in the findings

The final synthesized findings will be graded according to the ConQual approach for establishing confidence in the output of qualitative research synthesis and presented in a Summary of Findings.^24^ The Summary of Findings includes the major elements of the review and details of how the ConQual score was developed. Included in the Summary of Findings will be the title, population, phenomena of interest, and context for the specific review. Each synthesized finding from the review will then be presented, along with the type of research informing it, the score for dependability and credibility, and the overall ConQual score.

## Appendix I: Search strategy

### MEDLINE (Ovid)

Date searched: August 12, 2021
